# Concurrent acute pancreatitis and pericardial effusion

**DOI:** 10.11604/pamj.2015.21.122.6988

**Published:** 2015-06-15

**Authors:** Yusuf Kayar, Kenan Ahmet Turkdogan, Birol Baysal, Nigar Gultekin, Ahmet Danalioglu, Ali Tuzun Ince, Hakan Senturk

**Affiliations:** 1 Bezmialem Vakif University, Department of Internal Medicine, Division of Gastroenterology, Istanbul, Turkey; 2 Adnan Menderes University, Department of Emergency Medicine, Aydin, Turkey

**Keywords:** Acute pancreatitis, pericardial effusion, pleural effusion

## Abstract

While pleural effusion and ascites secondary to acute pancreatitis are common, clinically relevant pericardial effusion and cardiac tamponade are observed rarely. In a study by Pezzilli et al., pleural effusion was noted in 7 of the 21 patients with acute pancreatitis whereas the authors detected pericardial effusion development in only three. The authors asserted that pleural effusion was associated with severe acute pancreatitis, while pericardial effusion and the severity of acute pancreatitis were not significantly related.

## Introduction

Pleural effusion and ascites develop frequently in acute severe pancreatitis, while pericardial effusion does rarely [1, 2]. In patients with concurrent acute pancreatitis and pericardial effusion, development of pericardial effusion is generally associated with acute pancreatitis. In a case reported by Bastian et al., the authors did not detect amylases in pericardial fluid and therefore suggested that acute pancreatitis and pericardial effusion could be secondary to viral infection [3]. Herein, we present a patient who developed acute pericarditis and pericardial effusion concurrently with acute pancreatitis secondary to viral infection.

## Patient and observation

**Figure 1:**
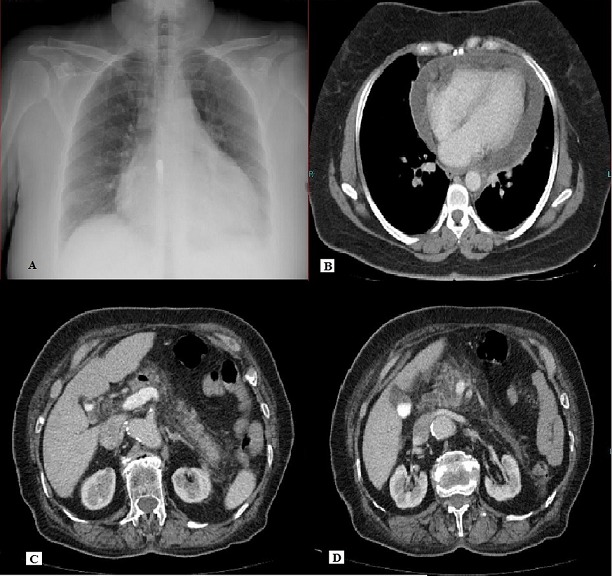
(A): chest X-ray on admission showing a massive enlargement of the cardiac silhouettewithout pleural effusions; (B): pericardial effusion reaching 23 mm at the thickest point with thoracic tomography; (C, D): pancreatic, peripancreatic inflammation and peripancreaticlocular fluidwith abdominal tomography

A 44-year-old female presented with cough, phlegm, nasal discharge and fever. A week later, she developed abdominal pain radiating to the back, chest pain and shortness of breath, therefore admitted for inpatient care. Her history included asthma and diabetes mellitus. Her familial history involved no relevant findings. The findings on physical examination were as follows: arterial blood pressure: 110/70 mm Hg, heart rate per minute: 102, fever: 38,2°C, oxygen saturation: 93% and tachypnea. Cardiac sounds were coming deeply and pericardial friction rubs were heard. Respiratory sounds were coarse bilaterally. Widespread abdominal tenderness was remarkable. Examinations of other systems yielded normal results. With laboratory analyses: leukocyte: 17,5 x109 /L, neutrophil: 41%, lymphcoyte: 53%, Hct: 36%, platelet: 359 x109 /L, CRP: 209 mg/L, amylase: 1552 IU/L, lipase: 1238 IU/L, AST: 24 IU/L, ALT: 28 IU/L, ALP: 68 IU/L, GGT: 28 IU/L, LDH: 232 IU/L, total bilirubin: 0,8µmol/L, conjugated bilirubin: 0,4µmol/L, glucose: 123 mg/dL, creatinine: 0,5 mg/dL. Other biochemical tests yielded normal results. Electrocardiogram demonstrated sinus tachycardia and reduction in voltage. Chest X-ray on admission showed a massive enlargement of the cardiac silhouette without pleural effusions (Figure 1 (A)) and echocardiography (ECO) showed pleural effusion of 21 mm in size at the widest point. Abdominal and thoracic tomography demonstrated pericardial effusion reaching 23 mm in size at the thickest point, multiple lymph nodes of 20x11 mm in size at the mediastinal and hilar regions, inflammation in the pancreas and peripancreatic fluid collection (Figure 1 (B,C,D)). Pericardial fluid aspirated transcutaneously was of serous appearance. Fluid analyse showed transudate character dominated with lymphocytes (670 leukocytes, 70% lymphocytes). Amylase or lipase was not detected in the fluid. Coxackie serology in serum was positive and enterovirus negative. However, coxaicke and enterovirus serology of the pericardial fluid were negative. ANA, ANCA, RF, mycoplasma IgM, chlamydia IgM were negative. HSV IgM, CMV IgM, EBV VCA IgM and TORCH infection were negative. Quantiferon and Wright tests yielded negative results. Magnetic Resonance Cholangiopancreatography and Endoscopic ultrasonography demonstrated no biliary pathology and fistula. The patient was started on ibuprofen, inhaled beta agonist, inhaledantiinflammatory agent and oxygen therapy. Patient's shortness of breath and abdominal pain improved during follow-up and control chest X-Ray demonstrated regression in pericardial effusion. The condition was considered to be secondary to viral infection because of elevated acute phase reactants, lymphocytosis, spontaneously resolved pericarditis, positive viral serology and exclusion of other possible causes of acute pancreatitis and pericarditis. Serum amylase and lipase levels returned to normal in addition to clinical resolving and the patient was discharged. Second look with ECO 2 weeks later confirmed the disappearing of pericardial effusion.

## Discussion

The mechanism of pericardial effusion in acute pancreatitis has not been elucidated completely but a number of theories have been suggested, including chemical pericarditis due to pancreatic enzymes carried by lymphatic vessels or available in the circulation, necrosis of vascular walls in fatty areas, necrosis of the subpericardial fat and fistulous connection between abdominal and pericardial cavities [4]. Mediastinal pseudocyst has been reported as the cause of cardiac tamponadein one report [5]. Chylous pericardial effusion has also been described in acute necrotising pancreatitis in a few cases [6]. Digestion of structural proteins by activated pancreatic enzymes might have disturbed the pancreatic duct, resulting in duct leakage. One may hypothesize that chemical irritation of the visceral and parietal pericardium by pancreatic enzymes may have initiated an inflammatory reaction, confirmed by elevations in the levels of inflammatory markers in the pericardial fluid [7]. While there are sporadic case reports of pericarditis and acute pancreatitis development secondary to viral infections, these can also occur simultaneously. Pleural effusion was not found in our patient who developed acute pancreatitis and pericardial effusion due to acute pericarditis. Because of positivity of viral serology, low level of amylase and lipase in the pericardial fluid and exclusion of other possible causes of pericarditis, concurrent acute pancreatitis and pericardial effusion secondary to viral infection was considered. Usually, pericardial effusion disappears spontaneously after clinical recovery and no specific treatment is required [7]. Likewise, all symptoms of our patient resolved in one week with symptomatic treatment only. Consequently pericardial effusion disappeared completely in two weeks.

## Conclusion

In conclusion pericardial effusion secondary to acute pancreatitis may develop via several mechanisms. Acute pancreatitis secondary to viral infection and pericardial effusion development should also be considered in the differential diagnosis. Complete resolution is usually achieved with symptomatic treatment in acute pancreatitis except for pericardial effusion associated with fistula presence.
